# Unraveling the Underlying Interaction Mechanism Between *Dabie bandavirus* and Innate Immune Response

**DOI:** 10.3389/fimmu.2021.676861

**Published:** 2021-05-27

**Authors:** Chuan-min Zhou, Xue-jie Yu

**Affiliations:** ^1^ State Key Laboratory of Virology, School of Health Sciences, Wuhan University, Wuhan, China; ^2^ Department of Infectious Diseases, Zhongnan Hospital of Wuhan University, Wuhan, China

**Keywords:** *Bandavirus*, immune escape, type I interferon, SFTSV, PRRs

## Abstract

The genus *Bandavirus* consists of seven tick-borne bunyaviruses, among which four are known to infect humans. *Dabie bandavirus*, severe fever with thrombocytopenia syndrome virus (SFTSV), poses serious threats to public health worldwide. SFTSV is a tick-borne virus mainly reported in China, South Korea, and Japan with a mortality rate of up to 30%. To date, most immunology-related studies focused on the antagonistic role of SFTSV non-structural protein (NSs) in sequestering RIG-I-like-receptors (RLRs)-mediated type I interferon (IFN) induction and type I IFN mediated signaling pathway. It is still elusive whether the interaction of SFTSV and other conserved innate immune responses exists. As of now, no specific vaccines or therapeutics are approved for SFTSV prevention or treatments respectively, in part due to a lack of comprehensive understanding of the molecular interactions occurring between SFTSV and hosts. Hence, it is necessary to fully understand the host-virus interactions including antiviral responses and viral evasion mechanisms. In this review, we highlight the recent progress in understanding the pathogenesis of SFTS and speculate underlying novel mechanisms in response to SFTSV infection.

## Introduction

According to the International Committee on Taxonomy of Viruses (ICTV) (1), the genus *Bandavirus*, classified under the order *Bunyavirales*, family *Phenuiviridae*, comprises 7 species, including *Dabie bandavirus*, *Bhanja bandavirus*, *Guertu bandavirus*, *Heartland bandavirus*, *Hunter Island bandavirus*, *Kismaayo bandavirus*, and *Lone Star bandavirus*, which are represented by *Dabie bandavirus* (SFTSV), Bhanja virus (BHAV), Guertu virus (GTV), Heartland virus (HRTV), Hunter island virus (HUIV), Kismaayo virus (KISV), and Lone star virus (LSV) respectively.

*Dabie bandavirus*, known as severe fever with thrombocytopenia syndrome virus, is a negative-sense single-strand RNA virus that was first discovered in 2009 and mainly reported in China, South Korea, Japan, and most recently in Vietnam, Thailand, and Pakistan (2–7). SFTSV is transmitted through tick-bites (like reported *Haemaphysalis longicornis* and *Dermacentor silvarum*) and occasionally from person to person *via* contacting the blood of SFTS patients directly (8–11). *Haemaphysalis longicornis* was reported in Australia, the Pacific regions, and recently in the United States (12, 13), indicating transmission risks of SFTSV worldwide. SFTSV causes an emerging hemorrhagic fever disease, termed severe fever with thrombocytopenia syndrome (SFTS), according to its primary symptoms, including fever, thrombocytopenia, and leukocytopenia. Other clinical symptoms of SFTS include anorexia, fatigue, nausea, abdominal pain, vomiting, malaise, diarrhea (2). In 2018, the World Health Organization (WHO) declared SFTS as a priority disease. Many studies investigated the therapeutic or prevention strategies of SFTSV with optimistic results, including vaccine, neutralizing antibody, and antiviral agent (14–19).

**Figure 1:**
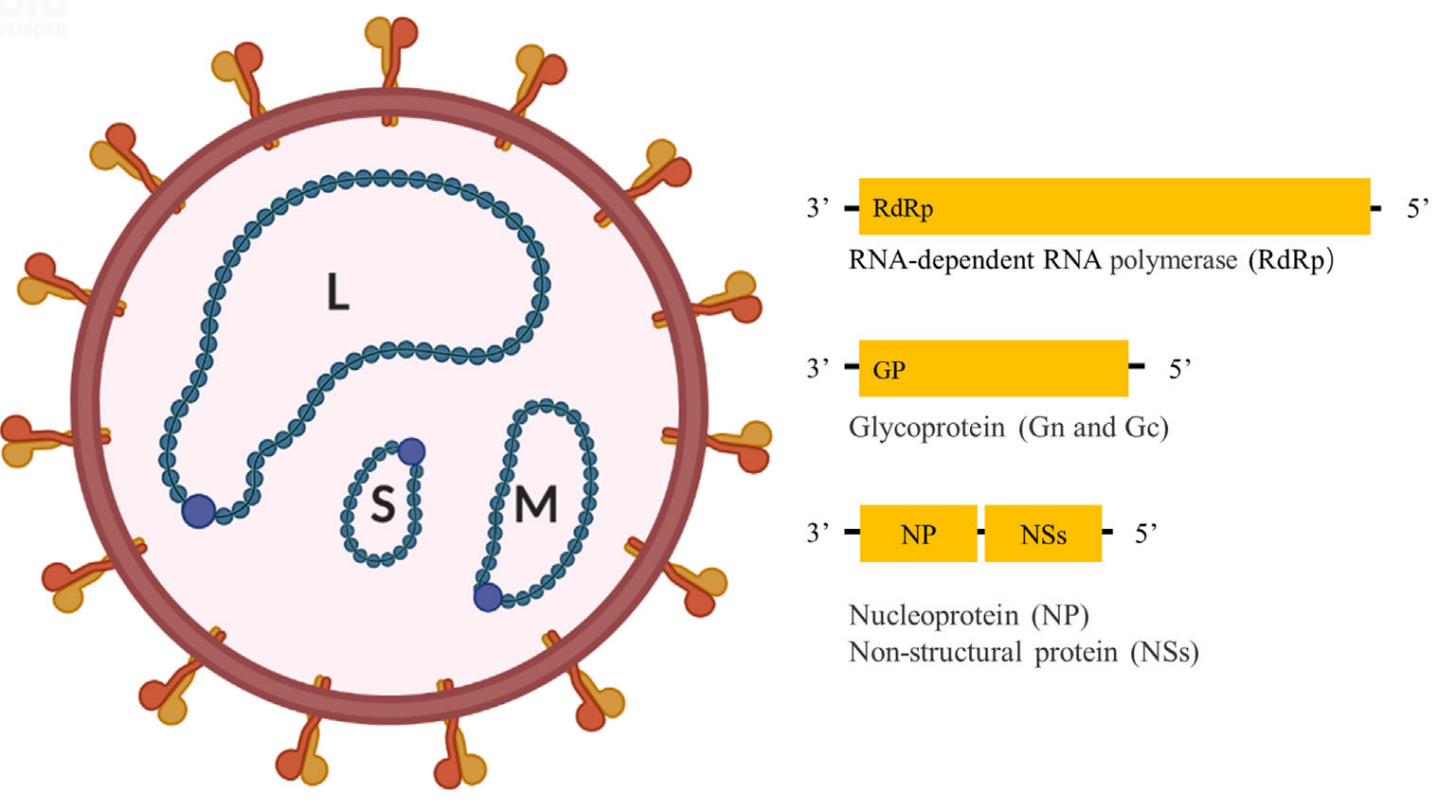
Schematic diagram of SFTSV structure.

Like other bandaviruses, SFTSV has a tripartite RNA genome consisting of Large (L), Medium (M), and Small (S) segments **(** **Figure 1** **)**; of which the L and M segments are encoded in the negative-sense orientation, while the S segment is encoded in an ambisense manner. The L segment encodes the RNA-dependent RNA polymerase (RdRp), mediating the replication of the viral genome. The M segment encodes glycoprotein (Gn and Gc) precursor that is cleaved into two glycoproteins, Gn and Gc, mediating virion assembly and viral entry into cells. The S segment encodes a non-structural protein (NSs) in the sense orientation and the nucleoprotein (NP) in the antisense orientation, serving as an important virulence factor and mediating genome replication and virion assembly respectively (2).

The innate immune response is the first line of host defense, mainly dependent on pattern recognition receptors (PRRs). Nucleic acids generated from viruses are one of the typical pathogen-associated molecular patterns (PAMPs), which could be specifically targeted by different nucleic acid pattern recognition receptors (PRRs). RIG-I-like-receptors (RLRs) are important in sensing RNA virus infections *via* recognition of viral RNA directly, such as eponymous retinoic acid-inducible gene 1 (RIG-I), mainly sensing short (<300 base pairs) dsRNA with a 5′triphosphate (5′ppp), which can be formed during SFTSV infection (20, 21). Activated RIG-I recruits mitochondria outer membrane protein MAVS and mediates downstream signaling cascade activation, leading to the phosphorylation and nuclear translocation of interferon-regulatory factors (IRFs) and nuclear factor kappa-light-chain-enhancer of activated B cells (NF-κB) and triggering the production of type I interferon (IFN). Type I IFNs bind to interferon-α/β receptor (IFNAR) mediating the activation of STAT1 and STAT2 heterodimers and resulting in transcriptional activation of IFN-stimulated genes (ISGs), which are crucial arms in the host defense system against viral challenges (22). Accordingly, SFTSV NSs can form inclusion bodies (IBs, also known as viroplasm-like structures) in SFTSV infected and NSs transfected cells. IBs colocalized with lipid droplets, and fatty acid biosynthesis was positively involved in the IBs formation and SFTSV replication (23). SFTSV NSs served as virulence molecules by capturing multiple host molecules into IBs, such as RIG-I, TBK1, IKK, IRF3, IRF7, TRIM25, STAT1, and STAT2 (21, 24–27), which were systematically introduced and discussed in several literatures (28, 29). Here, we summarize the current knowledge of innate responses and make hypotheses for the host-virus interaction mechanisms under SFTSV infection status.

## Autophagy in SFTSV Replication

Autophagy is an evolutionarily conserved catabolic process that is essential for maintaining cellular homeostasis. In general, autophagic pathways consist of three main routes: microautophagy, chaperone-mediated autophagy (CMA), and macroautophagy (hereafter referred to as autophagy). Of which, microautophagy is involved in taking up cytosolic material directly *via* lysosome membrane invagination, CMA refers to take up substrate proteins with KFERQ-motif selectively *via* lysosome-associated membrane protein type 2A (LAMP-2A), while autophagy is the best characterized one, involved in engulfing cellular materials into double-membrane vesicles for lysosome degradation. To date, multiple studies have clarified the complicated interconnections of the viral life cycle and the autophagic process (30, 31). Typically, autophagy is considered as an anti-viral response by degrading viral particles, enhancing viral antigen presentation, or involved in inflammatory and non-inflammatory response modulation. However, autophagy can be sometimes subverted or hijacked at different stages by certain viruses for viral assemble, replication, or even exocytosis.

To date, it is still poorly understood how *Bunyavirales* affected the host autophagy process and the role of autophagy in *Bunyavirales* infection. Canonical TLRs are reported to participate autophagy process in macrophages. TLR7 but not other TLRs might interact with and recognize RVFV glycoprotein directly (32). RVFV infection induced autophagy flux *via* the TLR7-MyD88 axis (33). Inhibition of multiple autophagy stages, from autophagic preinitiation to elongation phase, blocked RVFV infection, revealing a critical role for canonical autophagy in anti-RVFV response. Another study indicated that the Hantaan virus (HTNV), which belongs to the genus *Orthohantavirus*, induced mitophagy at the early infection stage but incomplete autophagy flux at the late infection stage for viral benefit (34). Autophagy is complexed and different viral proteins might exert reversed or synergic roles on the autophagic process (35, 36). In the early stage of HNTV infection, mitophagy was induced by HTNV Gn *via* interacting with mitochondrial protein TUFM, which inhibited MAVS-associated IFN response *via* degrading MAVS and therefore provided beneficial conditions for early-stage of HNTV replication. In the late stage of HNTV infection, the induction of autophagy was reversed by NP *via* disturbing Gn-LC3 interaction and interacting with SNAP29 for preventing autophagosome induction and autophagosome-lysosome formation respectively, which promoted viral replication and exocytosis.

As for SFTSV, limited studies investigated the underlying interaction of SFTSV and autophagy. SFTSV infection promoted the conversion of LC3-I to LC3-II in Vero cells, while lysosomal proteases inhibitor E64d and pepstatin A treatment did not affect the accumulation of LC3-II (37). LC3 deficiency was then found essentially for restricting SFTSV replication. Hence, based on the current information, SFTSV might restrict the autophagic degradation phase for viral replication. An alternative underlying mechanism is that SFTSV might be capable of inducing the occurrence of autophagy but blocking the autophagy flux. However, the amount and quality of data of SFTSV and autophagy was limited and not overwhelming (37), more autophagy-related experiments will be necessary to fully solidify the interaction of autophagy and SFTSV infection, such as detecting adaptor protein p62 and autophagy process by transmission electron microscopy (TEM).

SFTSV NSs exhibited colocalization with LC3, p62, and Lamp2b under SFTSV infection status, while SFTSV NSs alone was incapable of colocalizing with LC3, p62, and Lamp2b under NSs transfection status (37), indicating that other SFTSV proteins, such as RdRp, Gn, Gc, or NP, or host molecules may mediate this binding process. Besides, deletion of *atg7* cannot block the colocalization of SFTSV NSs and LC3, indicating that SFTSV NSs induced IBs were not conventional autophagosomes (38). It was worth mentioning that a portion of SFTSV NP was detected in the insoluble fraction and SFTSV NP was then shown interacting with SFTSV NSs protein directly (23), indicating that SFTSV NP but not NSs may be involved in the autophagy process under SFTSV infection, which will be worth for further investigation.

## Unfolded Protein Response and Mitochondrion Status During SFTSV Infection

Unfolded protein response (UPR) is an evolutionarily conserved signaling pathway, induced by ER stress, which plays a critical role in sensing and removing the accumulation of misfolded proteins and restoring homeostasis (39, 40). The UPR is mediated by three ER lumen transmembrane proteins and divided into three signaling branches, namely activating transcription factor 6 (ATF6), protein kinase (PKR)-like ER kinase (PERK), and inositol-requiring enzyme 1 (IRE1). Under a resting state, those transmembrane proteins are associated with glucose-regulated protein 78 (BiP). Under ER stress status, increased unfolded or misfolded proteins in the ER lumen promote the dissociation of BiP from those transmembrane proteins to elicit the UPR process. Like autophagy, UPR plays a double-edged role in response to various virus infections, either promoting virus elimination or subverted for virus invasion, indicating the intimate interaction between host UPR and viral life cycle.

**Figure 2:**
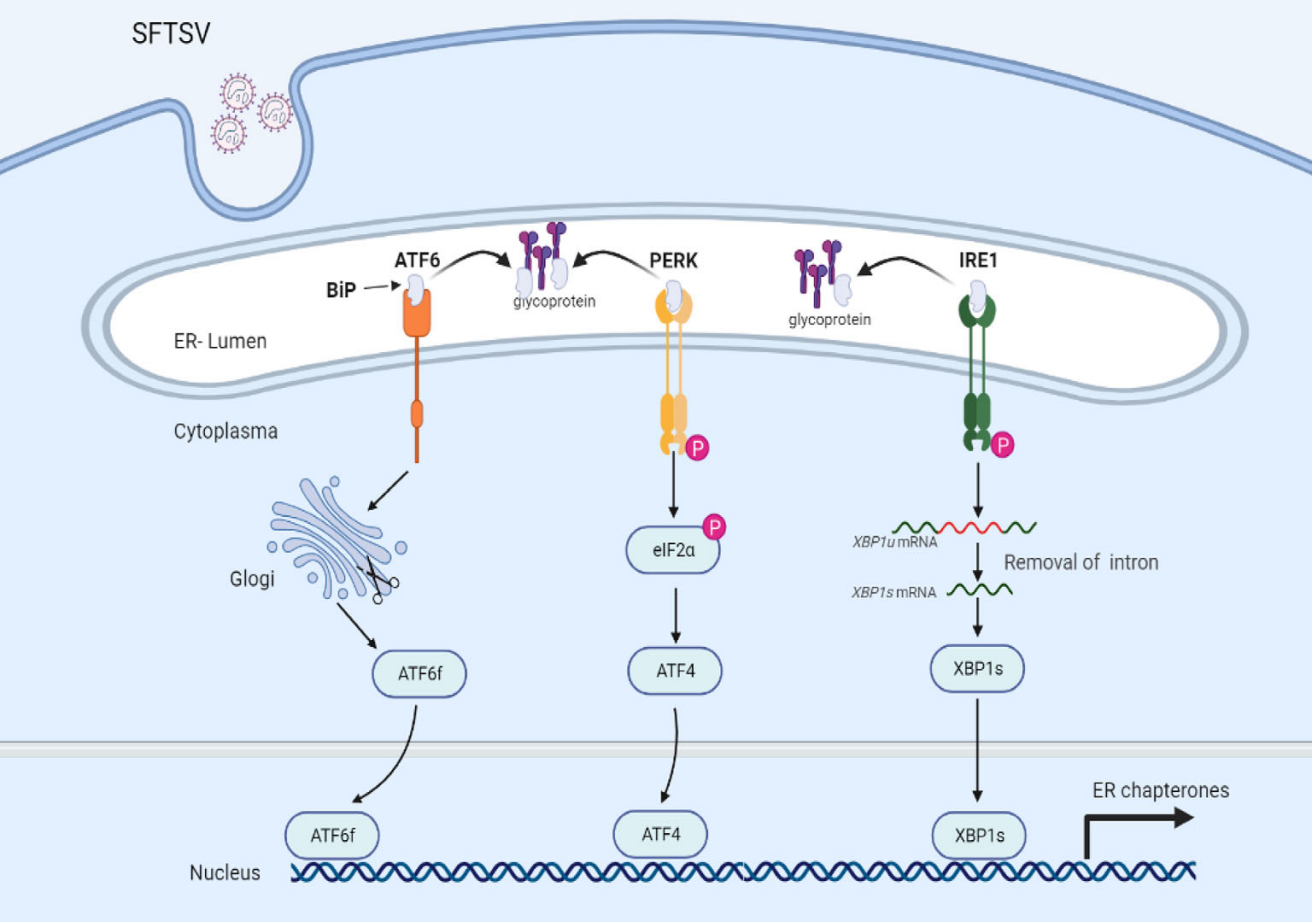
UPR is mediated *via* SFTSV infection. Under a resting state, transmembrane proteins (ATF6, PERK, and IRE1) are bound with glucose-regulated protein 78 (BiP) maintaining inactive status. Upon SFTSV infection, SFTSV GP mediated the disassociation of BiP with transmembrane proteins, leading to the activation of UPR.

To date, the detailed interactions between bunyaviruses and UPR have not been largely investigated. A recent study indicated that SFTSV infection could activate all three branches of UPR, which also occurred under HRTV and GTV infections. However, only PERK and ATF6, but not IRE1 were involved in favoring SFTSV replication. Further study indicated that GP **(** **Figure 2** **)**, but not other viral proteins (RdRp, NP, or NSs) of SFTSV, served as the inducer of the UPR (41). GP localizes to the endoplasmic reticulum-Golgi intermediate compartment (ERGIC) and Golgi complex (42). The assembly and exocytosis process of SFTSV virions occurs *via* Golgi apparatus and Golgi-derived vesicles respectively (43, 44). Hence, activation of the UPR may be induced by the accumulation of unfolded or misfolded proteins in the ER. SFTSV GP can be cleaved into Gn and Gc two subunits. However, it remains elusive whether Gn or Gc play any role in the UPR activation process.

In recent years, many studies have revealed that UPR pathways are connected to the autophagy activation process and many UPR-related transcription factors participate in autophagy activation by regulating the expression of autophagy-related genes (45). Despite no evidence indicating the interaction of SFTSV-GP and UPR, it is reasonable to propose the interaction between UPR pathways and the autophagic responses under SFTSV infection or SFTSV GP overexpression status. However, the role of UPR and autophagy in modulating SFTSV replication appears controversial. SFTSV replication induced by UPR might be independent of autophagy under SFTSV infection (37, 41). Overexpression of HTNV Gn was capable of promoting mitophagy, while NP inhibited Gn-mediated autophagy flux by competing with Gn for binding to LC3 (34, 46). Hence, it will be interesting to elucidate whether SFTSV- and GP-induced autophagy is associated with UPR.

UPR branches are well known for interacting with inflammatory mediators and triggering the downstream inflammatory pathways through various mechanisms. The complicated interaction between UPR and inflammation is relevant with multiple diseases, such as inflammatory bowel disease, cancer, and neurodegenerative diseases, which is systematically introduced in a recently published literature (47). Previous studies indicated that cytokine storm is associated with SFTS (48). Importantly, apart from IFNs, inflammatory cytokines are essential for innate immune response to some content against virus infection. However, dysregulated or excessive inflammation is harmful and associated with fatal consequences. Hence, it is worth to investigate whether SFTSV-induced inflammation is relevant to UPR.

**Figure 3:**
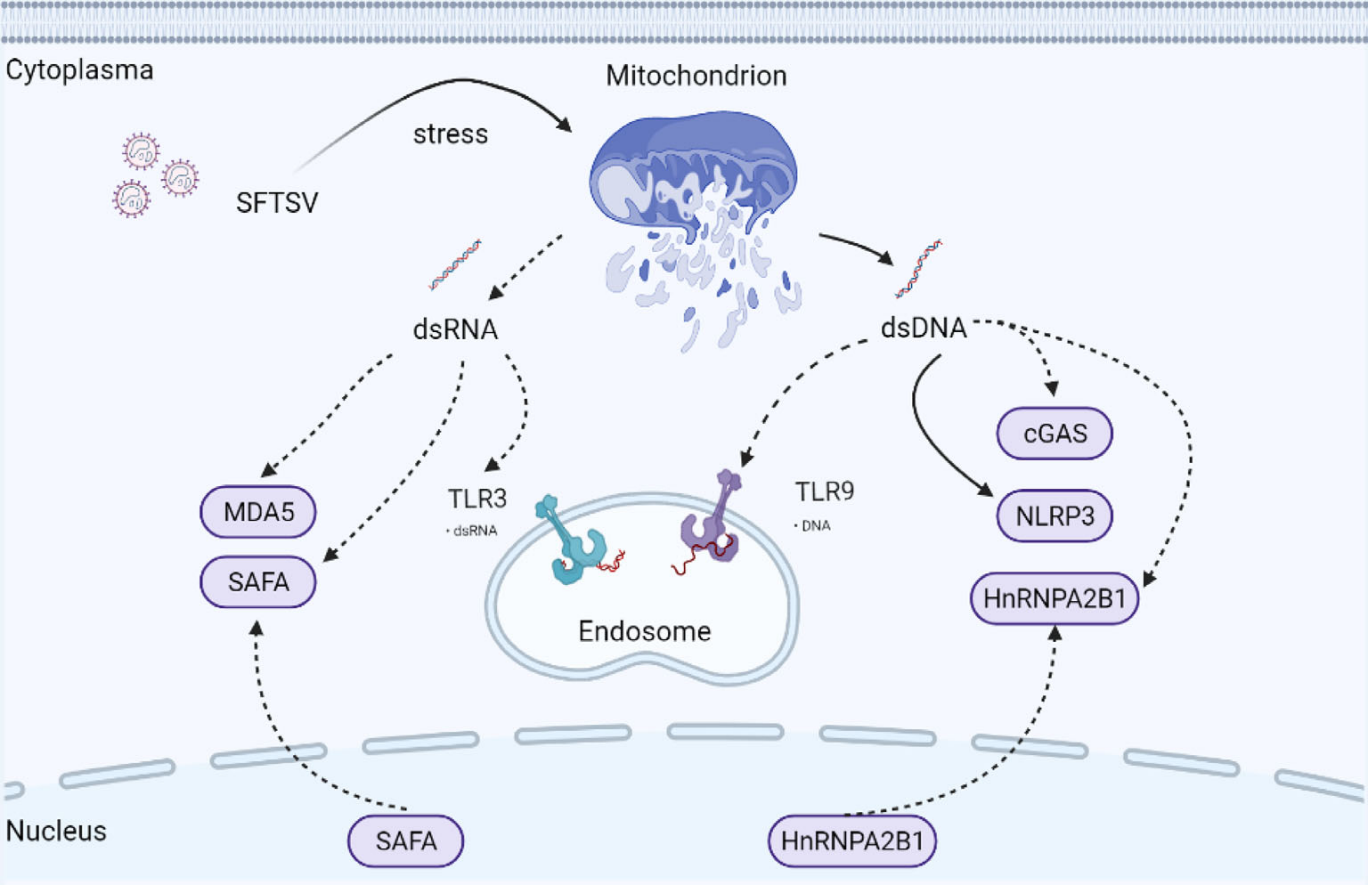
Possible underlying interaction of mitochondria and PRRs under SFTSV infection. Mitochondria are ubiquitous cellular organelles, orchestrating multiple biological processes. Under mitochondrion stress status, BAX/BAK axis is activated mediating mitochondrial permeabilization. mtdsDNA and mtdsRNA are then released into the cytoplasm, which triggers the activation of cytosolic PRRs mediated innate immune response. Solid arrows indicate confirmed mechanisms under SFTSV infection. Dotted arrows indicate unconfirmed or proposed mechanisms under SFTSV infection.

Mitochondria are ubiquitous cellular organelles, orchestrating multiple biological processes ranging from metabolism to programmed cell death (PCD) (49). Compared with DNA in the nucleus, the cell has a limited capacity to repair mitochondrial DNA (mtDNA). It has been reported for the first time that mtDNA can induce inflammation and arthritis in 2004 (50). To date, many studies have confirmed that mitochondria are critical in mediating host defense against virus infection (51–54). Mitochondria and mtDNA could be transferred to neighboring cells *via* connexin 43 gap junctions, tunnelling nanotubes, or exosomes (55–57). Under mitochondrion stress status, the mitochondrion is typically combined with ROS accumulation and release of mitochondrial components into the cytoplasm, such as cytochrome C and mtDNA, which is dependent on the pro-apoptotic BAK-BAX axis induced inner membrane herniation and mitochondrial outer membrane permeabilization (58). mtDNA is a circular dsDNA molecular harboring unmethylated CpG motifs similar to bacterial DNA, which mainly exists in the mitochondrial matrix. mtDNA is now accepted as a stimulator of the host immune system **(** **Figure 3** **)**.

Remarkably, mitochondrial genome transcription produces mitochondrial dsRNA (mtdsRNA), which can be degraded by mtdsRNA enzyme complexes RNA helicase SUV3 and polynucleotide phosphorylase (PNPase). Recently, mtdsRNA was found serving as a novel DAMP, initiating type I IFN response *via* classical dsRNA sensor melanoma differentiation-associated gene 5 (MDA5) **(** **Figure 3** **)**. SUV3 and PNPase deficiency were found to dampen the degradation of mtdsRNA (59). Also, BAK-BAX axis-induced macropores were important for mtdsRNA translocation from mitochondria into the cytoplasm (59). p53 deficiency promoted the accumulation of mtdsRNA in the cytoplasm, which can be recognized by RIG-I after cleaved by RNase L (60).

SFTSV infection disturbed mitochondrial homeostasis, induced mitochondrial damage, and promoted mitochondrial DNA oxidization and cytosolic release (61). We may ask whether mtdsRNA homeostasis is also disturbed under the SFTSV challenge. Previous studies indicated that RIG-I and MDA-5 were both upregulated under SFTSV infection (62, 63). RIG-I was involved in the recognition of SFTSV infection by sensing SFTSV RNA directly, while MDA5 was mainly sensing long dsRNA. Hence, it is interesting to explore the underlying mechanism of whether SFTSV infection would promote mtdsRNA accumulation and activate RIG-I and MDA5 dependent antiviral response.

Apart from those classical cytosolic RNA sensors like RIG-I and MDA5, Toll-like receptors (TLRs), including RNA sensor TLR3, TLR7, and TLR8, are located in endosomes, of which TLR3 senses dsRNA, whereas TLR7 and TLR8 are activated by ssRNA. Activated TLR3 mediates the activation of IRF3 and NF-κB *via* TRIF, while TLR7 and TLR8 utilize the adaptor MyD88 to activate IRF3, IRF5, IRF7, and NF-κB. Of those TLRs in endosomes, TLR3 was recently found capable of recognizing alcohol-associated stress-induced mtdsRNA accumulation (64). TLR3 expression was upregulated significantly compared with TLR-7 and TLR-8 under SFTSV infection in HepG2 cells (63). However, another study found that SFTSV suppressed the expression of TLR3 in both monocytes and myeloid dendritic cells (65). Hence, it is interesting to investigate the detailed role of TLR3 in different cell types responding to the SFTSV infection.

## Pyroptosis in Anti-SFTSV Immunity

Previous studies showed that SFTSV patients exhibited elevated levels of pro-inflammatory cytokines (48, 66), such as interleukin-1β (IL-1β), and cytokine storms could serve as biomarkers of SFTS patients. It should be noted that pro-inflammatory cytokines constitute a double-edged sword. On one hand, proper pro-inflammatory cytokines are key mediators of the innate immune system, playing an important role in host defense against virus attack. On the other hand, severe cytokine storms cause severe tissue damage in patients. In the context of the normal innate immune system, a well-coordinated and rapid innate immune response is the first line of defense against viral infection. Among those known PRRs, inflammasomes, including NLRs (NLRP3, and NLRC4) and ALRs (AIM2 and IFI16), play an important role in modulating the inflammatory response and cell pyroptosis. Typically, inflammasomes are a group of multi-protein platforms, containing inflammasome-initiating sensors, adaptor protein ASC, and pro-caspase-1, which are important for recognizing cellular damage- or pathogens-derived signals and orchestrating host defense against pathogen invasion (67). It is worth mentioning that NLRP3 inflammasome is the best-characterized inflammasome and to be associated with inflammation under RNA virus infection (68). NLRP3 inflammasome activation is divided into priming and maturation processes. Briefly, the TLR-NF-κB axis enhances the transcription of *pro-Il-1β* and inflammasome-associated components. Activated NLRP3 protein then recruits adaptor protein ASC and pro-caspase-1, inducing the pro-caspase-1 autoproteolytic cleavage into the active form caspase-1, promoting the maturation and secretion of pro-inflammatory cytokines IL-1β and IL-18 and driving gasdermin D (GSDMD)-dependent pyroptosis (69).

NLRP3 recognizes a variety of PAMPs and DAMPs, such as RNA, ATP, ionic flux, and lysosomal damage (69, 70). Importantly, mitochondria have diverse functions, ranging from energy conversion to maintaining cellular homeostasis. Accumulating data have indicated that mitochondria are central regulators triggering NLRP3 inflammasome-dependent antiviral immune responses and facilitating viral eradication (69, 71, 72). Mitochondria promote the NLRP3 inflammasome assembly *via* MAVS/Mfn2/MAM axis. The destabilization of mitochondria is believed to inducing NLRP3 inflammasome activation *via* ROS, Ca^2+^, NAD^+^, or mtDNA. Additionally, autophagy-mediated clearance of damaged mitochondria is confirmed prohibiting the activation of NLRP3 inflammasome (73).

Recent studies indicated strong NF-κB activation under SFTSV infection (61, 63, 74). The priming stage of inflammasome activation, including the transcription of *IL-1β*, is dependent on NF-κB activation. SFTSV infection activated BAX/BAK axis, reduced mitochondrial membrane potential, and induced mtROS production, strongly indicating that SFTSV infection caused dysfunction and damage of mitochondria (61). Generally, mitochondrial dysfunction is often associated with oxidization and the release of mtDNA into the cytosol. BAX/BAK mediated-permeabilization on the mitochondrial membrane is essential for the mitochondria-cytoplasm translocation of mtDNA (58). SFTSV infection was confirmed facilitating the maturation and secretion of pro-inflammatory cytokine IL-1β through NLRP3 inflammasome (61). NLRP3 mediated inflammatory response was associated with SFTSV fatal infection outcome. However, it remains elusive whether other inflammasomes are involved in recognizing SFTSV infection.

Similarly, the NLRP3 inflammasome is capable of recognizing RVFV and HNTV infection (75, 76). RVFV alone could barely promote the activation of the NLRP3 inflammasome. LPS-induced priming process was necessary for enhancing the activation of NLRP3 inflammasome under RVFV challenge, but not UV-RVFV. Unlike SFTSV, RVFV NSs was capable of forming filamentous structure in the nucleus, which was reported inducing transcriptional inhibition *via* interrupting of TFIIH complex assembly or promoting the degradation of TFIIH p62 subunit (77, 78). Enhanced production of IL-1β was expected in RVFV NSs deficient strain, indicating that RVFV NSs may be involved in immune escape of NLRP3 inflammasome (76). The priming stage of the inflammasome might be disturbed by RVFV NSs. Besides, both SFTSV NSs and HRTV NSs suppressed NLRP3 inflammasome-dependent IL-1β secretion (79). However, NSs of SFTSV promoted the activation of NF-κB (63, 74). Despite that NSs of SFTSV, HRTV, and RVFV were shown to restrict NLRP3-mediated antiviral immune responses, the detailed mechanism is still elusive. Many questions remain to be addressed. What is the conserved domain of NSs in restricting NLRP3 inflammasome activation? What are the NSs targeted inflammasome-related proteins?

## Possible Interaction of SFTSV With Novel Nucleic Acid Sensors

Apart from NLRP3, mtDNA interacts closely with multiple additional PRRs, including cyclic GMP-AMP synthase (cGAS), TLR9, NLRC4, and AIM2 (80, 81). cGAS is a well-characterized cytosolic DNA sensor catalyzing the generation of the nucleotide second messenger cyclic AMP-GMP (cGAMP), which then activates the stimulator of interferon gene (STING) (82, 83). Activated STING translocates from ER to ERGIC and Golgi, activating TBK1-IRF3 dependent IFN and proinflammatory responses (84), serving as the major defense mechanism against microbial infection. Several studies have indicated that cGAS is also responsible for sensing RNA virus challenges indirectly by recognizing mtDNA (51, 54). However, it is still elusive whether and how cGAS was activated by SFTSV. Now that mtDNA oxidization and cytosolic release were induced under SFTSV infection (61), it is reasonable to deduce that the cGAS-mediated IFN signal pathway could be activated by SFTSV-induced mtDNA release.

Apart from cellular viruses, many DNA viruses direct viral DNA into the nucleus. Accordingly, DNA sensors are also capable of existing and taking function in the nucleus, such as cGAS and IFI16 (85, 86). In 2019, Cao and coworkers confirmed a novel nuclear DNA sensor, namely heterogeneous nuclear ribonucleoprotein A2B1 (hnRNPA2B1), which is previously confirmed participating in RNA regulatory network (87–89). hnRNPA2B1 was activated and translocated from nucleus to cytoplasm, where it mediated cGAS-independent but STING-dependent innate immune response to DNA virus (90). It is well established that STING can be activated by bacterial cyclic dinucleotides (CDNs), such as cyclic di-AMP (c-di-AMP), as well as cGAMP. Phosphorylation of STING at Ser^366^ and Leu^374^ is important for the activation of STING (91, 92). However, it remains elusive how hnRNPA2B1 activates STING. Is hnRNPA2B1 participating in the phosphorylation of STING or promoting the translocating of STING? What is the conserved domain mediating the interaction of hnRNPA2B1 and STING?

hnRNPA2B1 was important for the innate immune-mediated inhibition of DNA virus replication, including HSV-1 and AdV, but not involved in the recognition of RNA virus VSV and SeV. Upon sensing viral DNA, nuclear-localized hnRNPA2B1 formed a complex with viral DNA and then formed a homodimer, which was then demethylated at arginine-226 by the arginine demethylase JMJD6. Mutation of the hnRNPA2B1 dimer interface was unable to associate with JMJD6 and abrogated hnRNPA2B1 nucleocytoplasmic translocation, inhibiting the subsequent initiation of the IFN-β signal pathway. Additionally, hnRNPA2B1 facilitated m^6^A modification and nucleocytoplasmic trafficking of mRNA *cGAS*, *IFI16*, and *STING* to amplify the activation of the IFN-β signal pathway in response to DNA virus infection. It remains unknown whether all RNA viruses not capable of activating hnRNPA2B1? Despite that hnRNPA2B1 mainly existed in the nucleus under HSV-1 infection, of interest to us is whether mtDNA could directly promote the activation of cytoplasmic hnRNPA2B1 or enhance the activation of nuclear hnRNPA2B1 under DNA virus infection.

In 2019, SAFA (nuclear scaffold attachment factor A) was identified as a novel nuclear dsRNA sensor (previously known as nuclear matrix protein hnRNPU) for both DNA and RNA viruses. Like hnRNPA2B1, SAFA was also a nucleic acid binding protein belonging to the hnRNP family. Upon sensing viral RNA in the nucleus, nuclear matrix protein SAFA oligomerized and promoted antiviral gene expression (93). Myeloid-specific SAFA-deficient mice showed decreased IFN-β activation, which was more susceptible and showed less resistance to HSV-1 and VSV infection. SAFA directly sensed viral RNA in the nucleus *via* its RGG domain and form homodimers. SAFA interacted with SMARCA5 and TOP1, two key components of the SWI/SNF nucleosome remodeling complex, *via* the middle SPRY domain leading to the transformation of chromosome conformation and activation of the enhancers and super-enhancers of antiviral genes.

The induction of SAFA by the virus was severely impaired in ELF4, STING, or MAVS deficient cells. Further studies indicated that ELF4 significantly promoted the expression of SAFA and the phenomena were further enhanced by STING and MAVS overexpression, indicating STING and MAVS may promote the expression of SAFA. The authors hypothesized that SAFA may take function through the STING or MAVS-dependent antiviral IFN pathway and was required for IRF3 and IRF7 recruitment to the IFN-β promoter. Based on the current information, SAFA took function in a cytoplasm-independent manner under VSV or HSV-1 infection, which cannot affect the activation and phosphorylation of IRF3. The detailed function of SAFA under virus infection is largely elusive. It will be an interesting topic to investigate whether SAFA can be translocated to cytoplasm like hnRNPA2B1 under infection status.

## Role of Apoptosis Under SFTSV Challenge

Apoptosis is a major form of regulated cell death, characterized by nuclear condensation, cell shrinkage, membrane blebbing, and DNA fragmentation (94, 95). Apoptosis is initiated through the extrinsic receptor-mediated pathway and intrinsic mitochondrial pathway. The extrinsic pathway is activated by extracellular stimulation *via* death receptors, such as TNF receptor, Fas, and TRAIL receptor. The intrinsic apoptotic pathway is activated by various intracellular stimuli, such as cytochrome c DNA damage, oxidative stress, and ER stress. Of note, mitochondria are crucial for the activation of apoptosis (96, 97). Under cellular stress conditions, the Bcl-2 effector proteins BAK/BAX cause mitochondrial membrane permeabilization, which in turn promotes the release of cytochrome c into cytoplasm, leading to the activation of apoptosis executioner caspase endopeptidases, caspase-3 and -7, cleaving hundreds of cell substrates, and inducing apoptosis. Additionally, upon apoptosis receptor activation, the intracellular receptor region recruits the adapter protein FADD, which in turn mediates the activation of caspases-3 and -7 induced apoptosis (98). Besides, the immune response induced by mtDNA leakage is partially prevented by the activation of apoptosis and related caspase activity, including caspase-3, -7, and -9. Mitochondrion-related caspases are shown cleaving or inhibiting the activation of multiple PRRs directly, such as cGAS, STING, IRF3, or MAVS (99–101).

Apoptosis is a highly regulated process, eliciting both negative and positive effects on the viral life cycle. On one hand, apoptosis is involved in eliminating virus-infected cells. On the other hand, viruses, mainly through viral proteins, might take advantage of apoptosis for exocytosis *via* regulating the expression and function of death receptors, caspase endopeptidases, or apoptotic proteins (102, 103). Previous studies indicated that apoptosis was not or slightly induced under SFTSV infection in monocytes (61, 62), and this was supported by SFTSV related proteomic analysis, which revealed that several proteins with anti-apoptosis roles were upregulated under SFTSV infection, such as SOD2, BCL3, CD74, FAM129B (61). Apoptosis may be involved in SFTSV-associated immunopathology. However, the detailed mechanisms remain unclear and further study will be necessary to investigate the balance mechanism of apoptosis.

## Knowledge Gap

Currently, there is no vaccine or treatment available to prevent SFTS. SFTSV poses a serious threat to public health, while the pathogenic mechanism of SFTSV is largely elusive. The viral-mediated immune responses are complex processes. It is just a beginning to unravel the detailed interacted mechanisms of SFTSV in host defense. In this review, we have discussed the well-established role and highlighted the underlying mechanism of innate immunity in response to SFTSV infection. Several important questions remain to be answered: Are SFTSV viral components (not limited to NSs) taking part in the immune escape of those innate immune responses? How can SFTSV hijack the host process for viral replication? How can autophagy participate in sensing and defending SFTSV challenges? Is the UPR related to SFTSV-induced autophagy and inflammation? What are the detailed mechanisms of SFTSV in inducing mitochondrial stress? Are mitochondria playing a key role in host defense against SFTSV infections? Are mtdsRNA and mtDNA taking part in the modulation of classical RNA and DNA PRRs under the SFTSV challenge? Are the nucleic acid sensors such as SAFA or hnRNPA2B1 capable of sensing SFTSV infection? If so, what is the immune escape mechanism of SFTSV? Answering and discovering those possible interconnections will not only enhance our understanding of the viral mechanism to interact with host immunity but also lead to the potential anti-SFTSV strategies.

## Author Contributions

Conceptualization, C-mZ. Original draft preparation, C-mZ. Writing—review and editing, C-mZ and X-jY. Project administration, C-mZ and X-jY. Funding acquisition, C-mZ and X-jY. All authors contributed to the article and approved the submitted version.

## Funding

This study was supported by the National Natural Science Funds of China (81971939 and 31570167) and the Fundamental Research Funds for the Central Universities (2042021kf0046). The funders had no role in the study design, data collection and analysis, decision to publish, or the preparation of the manuscript.

## Conflict of Interest

The authors declare that the research was conducted in the absence of any commercial or financial relationships that could be construed as a potential conflict of interest.
